# Supplementary material for: Comprehensive characterization of claudin-low breast tumors reflects the impact of the cell-of-origin on cancer evolution
Source: Nat Commun. 2020 Jul 9;11:3431. doi: 10.1038/s41467-020-17249-7 (PMC7347884; doi:10.1038/s41467-020-17249-7)
Supplement: Supplementary file 1 — Supplementary Information [file 41467_2020_17249_MOESM1_ESM.pdf]

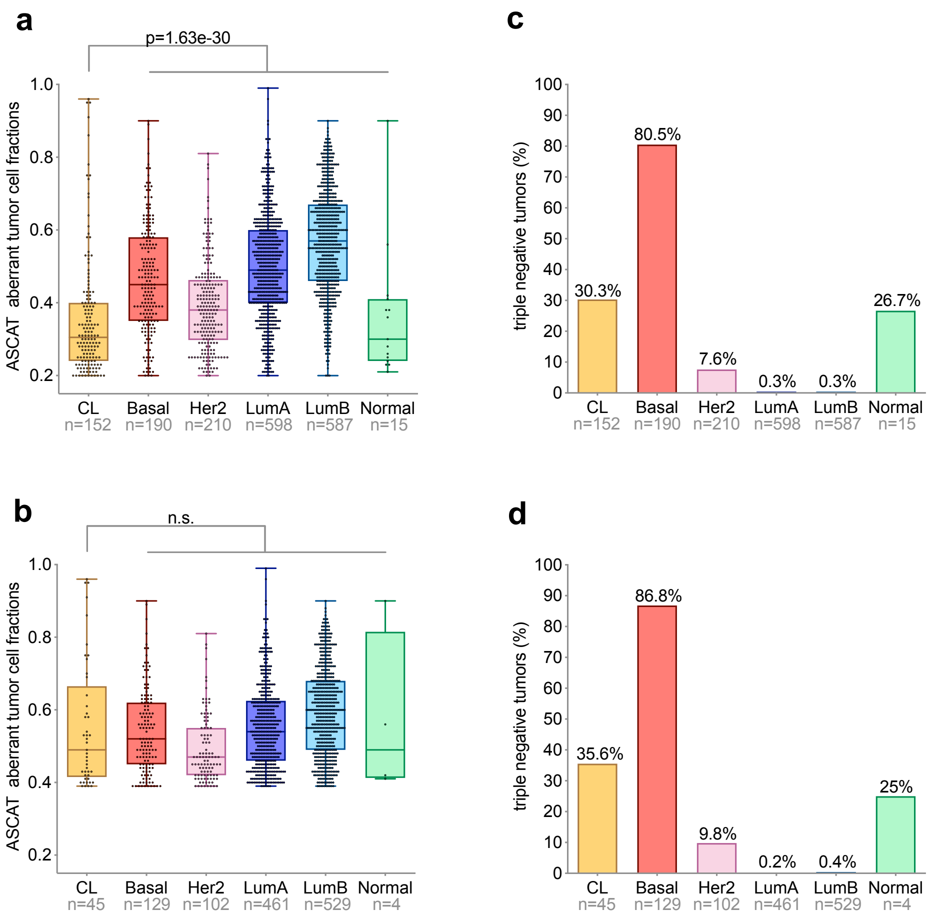

Supplementary Fig. 1: Selection of tumors according to their cellularity
a, b- Tumor cell fraction for each molecular subtype from the METABRIC cohort from ASCAT method **a-** before and **b-** after purity threshold filtering (ASCAT cancer cell fraction > 0.38). Wilcoxon test; all data points are shown. **c, d-** Percentage of triple negative breast tumors in each molecular subtype **c-** before and **d-** after purity threshold filtering (ASCAT cancer cell fraction > 0.38). Wilcoxon tests. Boxplot: center line, median; box limits, upper and lower quartiles; whiskers, minimum to maximum.

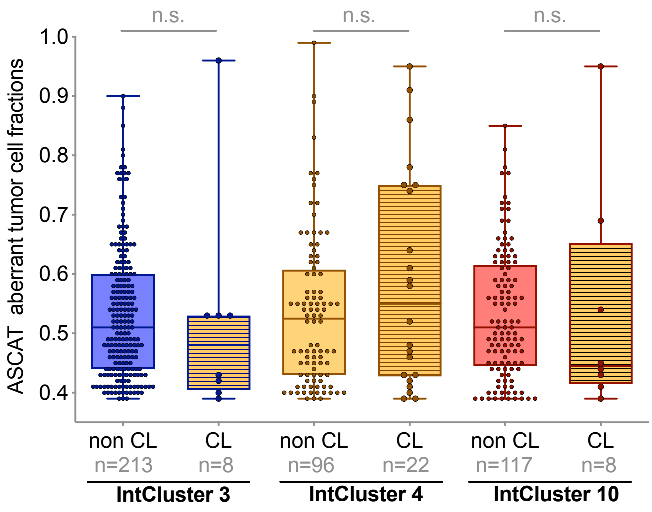

Supplementary Fig. 2: Tumor cell fraction (ASCAT method) for IntClusters 3, 4 and 10 tumors from the METABRIC cohort according to their Claudin-low status
Wilcoxon tests. Boxplot: center line, median; box limits, upper and lower quartiles; whiskers, minimum to maximum; all data points are shown.

a

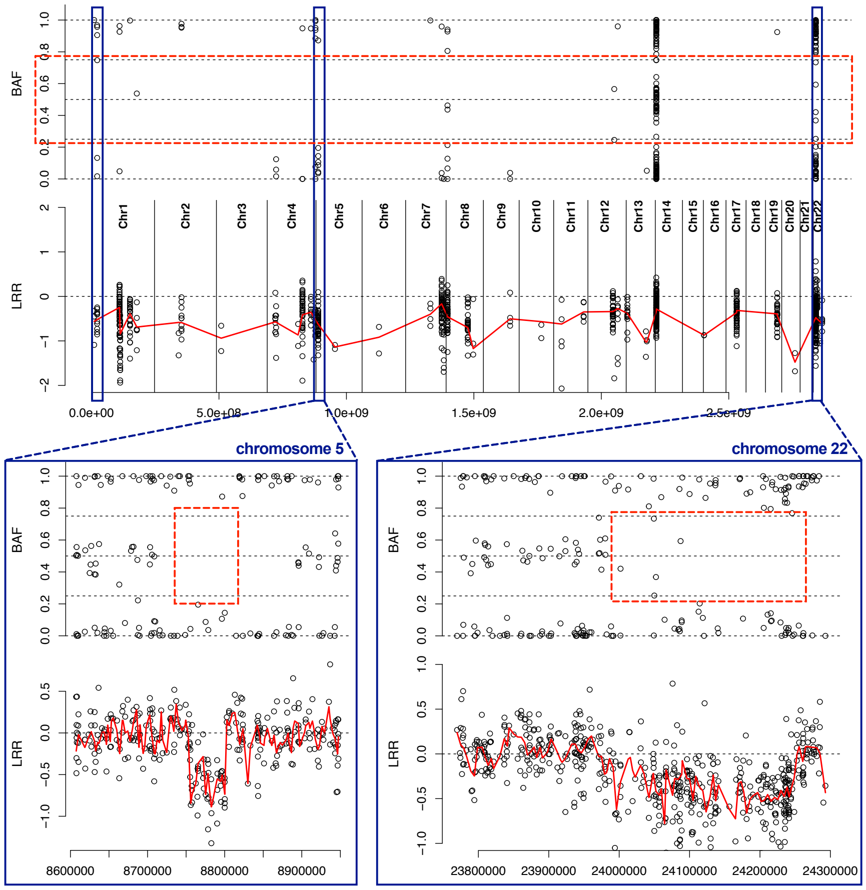

b

gene	mutation type	chromosome	exon	reference allele	altered allele	vaf	FGA (%)	ASCAT purity
**DNAH11**	missense SNV	chr7	exon23	A	G	0.47	0.70%	0.78
**NOTCH1**	missense SNV	chr9	exon13	G	T	0.57		
**ARID2**	missense SNV	chr12	exon15	C	T	0.48		
**TP53**	nonsense SNV	chr17	exon4	C	T	0.04		

c

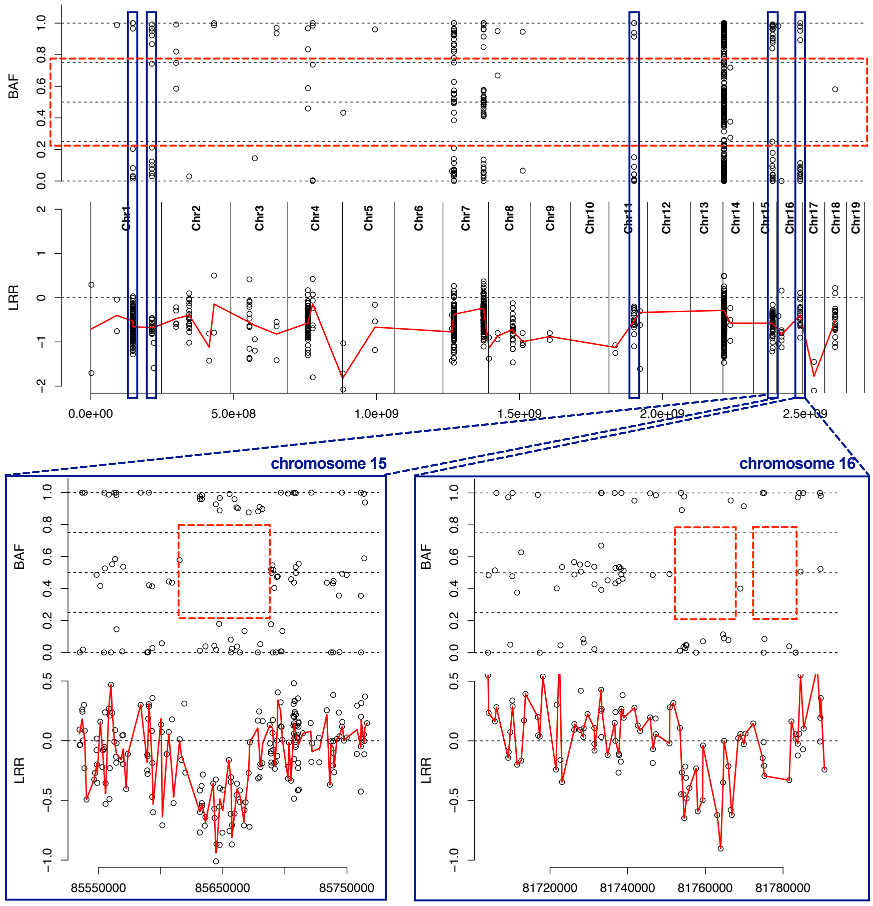

Supplementary Fig. 3: Integrative cluster 4 (IntClust4) claudin-low tumors display rare focal copy number alterations

Identification of focal genomic alterations in CNA-devoid IntClust4 claudin-low samples (**a-b-** MB-4711 / FGA < 0.7%; **c-** MB-0540 / FGA < 0.3%). **a, c-** BAF and LRR plots of SNPs localized in genomic regions of copy number deletion (LRR < 0.4). **c-** Mutation analysis of MB-4711 sequencing data. No somatic mutations were identified among the targeted sequenced genes from the MB-0540 sample.

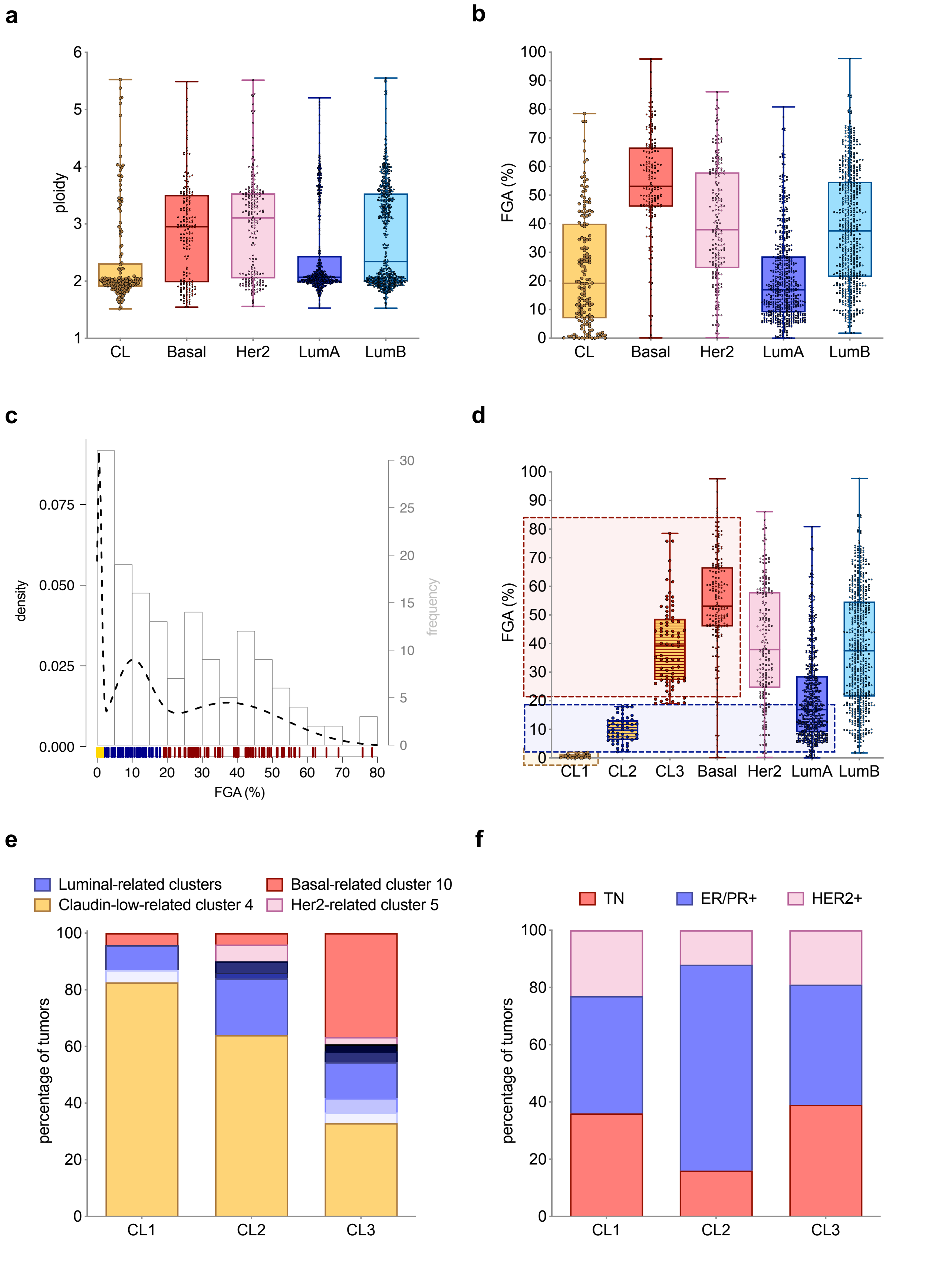

Supplementary Fig. 4: Genomic and molecular analyses of breast cancer samples from whole METABRIC cohort without tumor purity selection

a- Ploidy for each molecular subtype of the METABRIC cohort. **b-** FGA% for each molecular subtype. **c-** Gaussian mixture model application on FGA distribution across claudin-low tumors. **d-** FGA% in claudin-low subgroups compared to other molecular subtypes. **e-** Integrative clusters and **f-** breast cancer receptor status distribution in each claudin-low subgroup. Boxplot: center line, median; box limits, upper and lower quartiles; whiskers, minimum to maximum. CL: claudin-low; TN: triple negative. Claudin-low tumors (n=152); basal tumors (n=190); HER2 tumors (n=210); luminal A tumors (n=198); luminal B tumors (n=587); CL1 tumors (n=23); CL2 tumors (n=50); CL3 tumors (n=79). Each bar on the x axis corresponds to one claudin-low tumor.

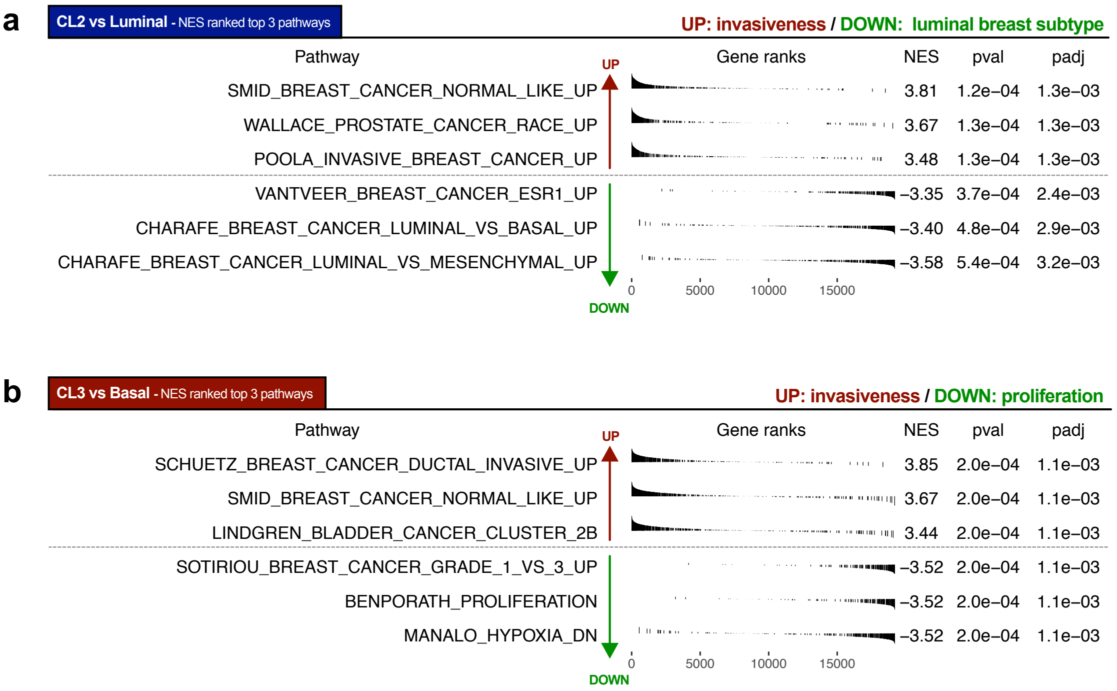

Supplementary Fig. 5: CL2 and CL3 tumors display attenuated transcriptomic features compared to their non-claudin-low counterparts

a-b- GSEA comparing global gene expression of METABRIC tumors from **a-** CL2 subgroup to the two luminal (luminal A and luminal B) subgroups, and from **b-** CL3 subgroup to the basal-like subgroup. The three pathways with the highest and lowest enrichment scores are represented (> 15,000 tested pathways – NES ranking). CL2 and CL3 subgroups show respectively lower luminal- and basal-related signature scores than luminal and basal-like breast tumors but present a significant enrichment in invasiveness-related signatures.

a

CL1 discriminant genes

GENE SYMBOL	ENTREZ_ID	CL1 centroids	CL2 centroids	CL3 centroids
ANKRD22	118932	-1.21	0.29	0.40
TIMM17B	10245	-1.13	0.27	0.37
KPTN	11133	-1.11	0.22	0.41
CXCL10	3627	-1.10	0.42	0.22
NEK2	4751	-1.09	0.36	0.26
PBK	55872	-1.08	0.25	0.36
CDS1	1040	-1.06	0.26	0.35
CENPF	1063	-1.06	0.17	0.42
ARHGAP39	80728	-1.06	0.21	0.39
C12orf66	144577	-1.06	0.26	0.34
ILDR1	286676	-1.04	0.25	0.34
PYGO2	90780	-1.03	0.16	0.42
EPCAM	4072	-1.03	0.29	0.30
MRPL58	3396	-1.02	0.27	0.31
TOP2A	7153	-1.01	0.06	0.50
TAF6	6878	-1.01	0.29	0.29
STAT1	6772	-1.01	0.46	0.12
MCM4	4173	-1.00	0.12	0.44
ISG15	9636	-1.00	0.28	0.29
FKBP4	2288	-1.00	0.29	0.28
GFPT1	2673	-0.99	0.44	0.13
UBE2T	29089	-0.99	0.28	0.29
KIAA0895	23366	-0.99	0.12	0.43
GRHL2	79977	-0.99	0.13	0.42
PLK4	10733	-0.98	0.11	0.44
MIS18A	54069	-0.98	0.06	0.49
GINS2	51659	-0.98	0.27	0.29
CCNB1	891	-0.98	0.27	0.29
CCDC8	83987	0.97	-0.45	-0.11
PRDM2	7799	0.98	-0.16	-0.39
PDSS2	57107	0.98	-0.44	-0.13
CCL21	6366	0.98	-0.17	-0.38
FEZ1	9638	0.98	-0.26	-0.30
CHST7	56548	0.98	-0.07	-0.48
SNCA	6622	0.99	-0.31	-0.26
ADAMTS5	11096	0.99	-0.34	-0.23
GRIA3	2892	0.99	-0.29	-0.28
ALPL	249	0.99	-0.39	-0.18
NAALADL1	10004	1.00	-0.25	-0.32
VWCE	220001	0.99	-0.47	-0.11
ADCY4	196883	1.00	-0.23	-0.34
NPHP3	27031	0.99	-0.44	-0.13
CAPN11	11131	1.00	-0.25	-0.32
FAM43A	131583	1.00	-0.19	-0.38
ECSCR	641700	1.00	-0.13	-0.43
FOXO1	2308	1.01	-0.35	-0.23
FAM66B	100128890	1.00	-0.15	-0.42
PTTG2	10744	1.00	-0.50	-0.08
CRTAP	10491	1.01	-0.40	-0.18
ANKRD29	147463	1.01	-0.23	-0.35
LRIG3	121227	1.00	-0.51	-0.07
PRDM16	63976	1.01	-0.46	-0.12
LINC00341	79686	1.01	-0.18	-0.39
FCER1A	2205	1.00	-0.06	-0.50
LAYN	143903	1.02	-0.39	-0.20
EEPD1	80820	1.01	-0.52	-0.06
KRTAP1-1	81851	1.02	-0.38	-0.21
CTSG	1511	1.02	-0.20	-0.38
IL11RA	3590	1.02	-0.34	-0.25
TGFBR2	7048	1.01	-0.03	-0.53
PROS1	5627	1.02	-0.32	-0.26
CCBE1	147372	1.02	-0.32	-0.27
AKAP13	11214	1.02	-0.48	-0.11
EFCAB1	79645	1.02	-0.46	-0.14
MRGPRF	116535	1.03	-0.39	-0.20
SVEP1	79987	1.02	-0.14	-0.44
MME	4311	1.03	-0.27	-0.31
KANK3	256949	1.03	-0.23	-0.35
SLC2A12	154091	1.03	-0.43	-0.16
FLRT2	23768	1.03	-0.25	-0.34
FAM107A	11170	1.04	-0.31	-0.29
GFRA2	2675	1.04	-0.23	-0.37
CCDC140	151278	1.04	-0.46	-0.14
ZHX3	23051	1.04	-0.44	-0.16
ELANE	1991	1.05	-0.29	-0.31
S1PR1	1901	1.03	0.02	-0.59
HBA2	3040	1.05	-0.20	-0.39
ADAMTSL4	54507	1.05	-0.48	-0.13
PDE2A	5138	1.06	-0.34	-0.27
EBF3	253738	1.06	-0.24	-0.37
ABCA6	23460	1.06	-0.16	-0.44
GPIHBP1	338328	1.06	-0.40	-0.21
CLEC3B	7123	1.06	-0.15	-0.45
CXCL5	6374	1.07	-0.35	-0.26
ZMAT3	64393	1.08	-0.13	-0.48
FAM13C	220965	1.09	-0.35	-0.28
FIGNL2	401720	1.10	-0.41	-0.22
CAVIN2	8436	1.10	-0.17	-0.45
ASIP	434	1.11	-0.27	-0.36
PTH1R	5745	1.09	-0.57	-0.07
CCL23	6368	1.12	-0.45	-0.19
TACC1	6867	1.12	-0.35	-0.29
DPT	1805	1.12	-0.38	-0.26
PID1	55022	1.15	-0.26	-0.39
KLHL3	26249	1.15	-0.30	-0.36
EPAS1	2034	1.16	-0.30	-0.36
CXCL12	6387	1.16	-0.38	-0.29
STAB2	55576	1.16	-0.41	-0.25
MMRN1	22915	1.17	-0.27	-0.40
CLDN5	7122	1.20	-0.31	-0.38
CYTL1	54360	1.23	-0.30	-0.40
SNX1	6642	1.23	-0.38	-0.33
ACVRL1	94	1.23	-0.44	-0.27
CD1C	911	1.23	-0.19	-0.51
PXK	54899	1.24	-0.52	-0.20
HOXD12	3238	1.29	-0.45	-0.29
LYVE1	10894	1.32	-0.50	-0.26

CL2 discriminant genes

C3orf52	79669	-0.74	0.80	-0.34
DNAJC12	56521	-0.61	0.73	-0.35
BIK	638	-0.76	0.72	-0.26
BUB3	9184	-0.46	0.69	-0.40
PVALB	5816	-0.54	0.69	-0.35
ISOC1	51015	-0.51	0.69	-0.37
LEO1	123169	-0.63	0.68	-0.29
TMEM209	84928	-0.80	0.67	-0.19
SPATA17	128153	-0.74	0.67	-0.22
FYB2	199920	-0.59	0.67	-0.30
HEPH	9843	0.56	-0.67	0.32
NLRX1	79671	0.85	-0.66	0.15
UBE2E2	7325	0.54	-0.67	0.33
CCDC102A	92922	0.59	-0.67	0.31
NXN	64359	0.60	-0.70	0.33
STOX2	56977	0.38	-0.72	0.46
OXT	5020	0.41	-0.76	0.49
MFGE8	4240	0.52	-0.77	0.44
PLD1	5337	1.01	-0.65	0.05

CL3 discriminant genes

TKTL2	84076	0.34	0.51	-0.67
ITPR1	3708	0.22	0.56	-0.66
ZNF205	7755	-0.63	-0.31	0.64
PSMD2	5708	-0.56	-0.35	0.64
DCBLD1	285761	-0.12	-0.60	0.64
ECE2	9718	-0.77	-0.22	0.63
KIF20A	10112	-0.81	-0.21	0.65
ZNF664	144348	-0.90	-0.16	0.65
ZSWIM4	65249	-0.30	-0.54	0.68
ACTN4	81	-0.24	-0.58	0.68
CLDN3	1365	-0.80	-0.32	0.74

b

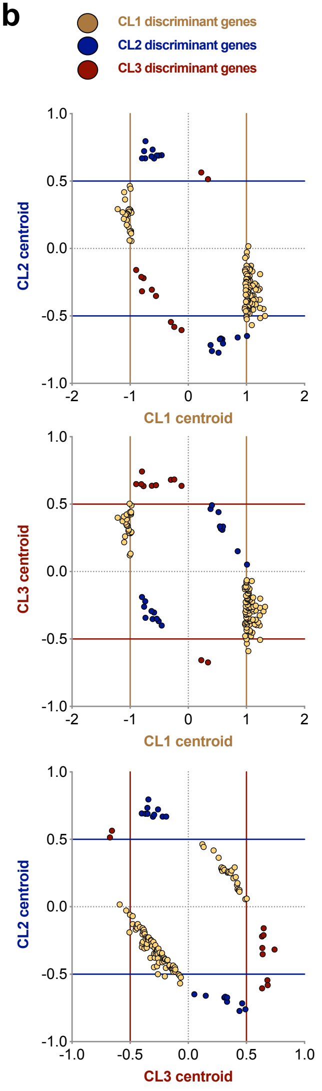

c

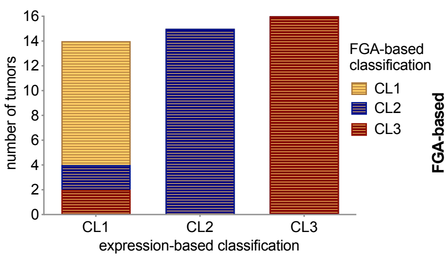

FGA-based classification \ expression-based classification	CL1	CL2	CL3
CL1	10	0	0
CL2	2	15	0
CL3	2	0	16

accuracy: 91.1%

d

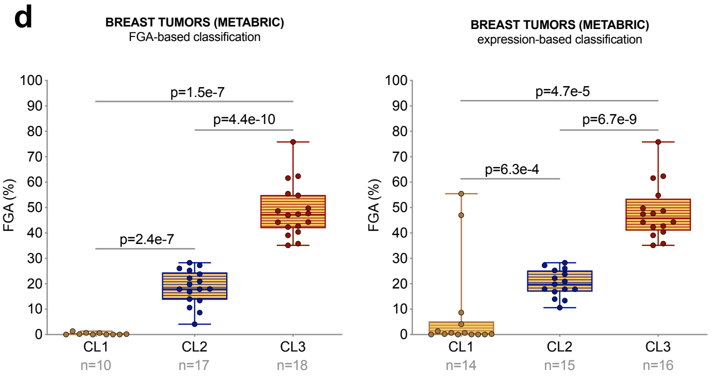

e

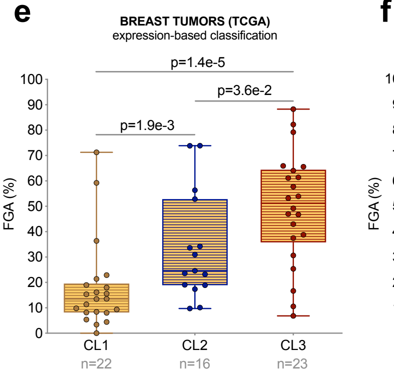

f

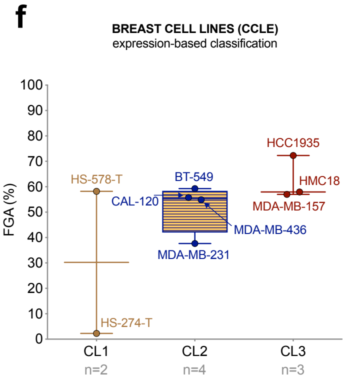

g

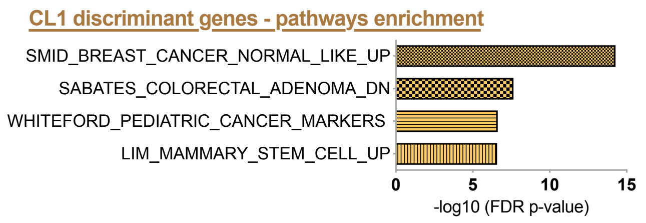

Supplementary Fig. 6: Generation of a gene-expression based classifier for identifying CNA-related claudin-low subgroups

a- Centroid-associated gene expression values in each claudin-low subgroup and **b-** representative scatterplots. CL1 discriminant genes are indicated in yellow, CL2 in blue and CL3 in red. **c-** Accuracy between claudin-low subgroup assignment from Gaussian mixture model (FGA-based classification) and centroid predictor (expression-based classification). **d-f-** FGA distribution for each claudin-low subgroup from **d-** METABRIC claudin-low tumors classified according to Gaussian mixture model on FGA distribution (left panel) or expression-based centroid method (right panel), **e-** TCGA claudin-low tumors and **f-** CCLE claudin-low cell lines classified according to expression-based centroid method. In all tumors and cell lines cohorts, CL1 tumors display very few CNA, CL2 tumors show intermediate level of FGA and CL3 present high degree of genomic alterations. Wilcoxon tests. **g-** Pathway enrichment analysis of CL1 discriminant gene list highlights stemness related pathways. Boxplot: center line, median; box limits, upper and lower quartiles; whiskers, minimum to maximum; all data points are shown.

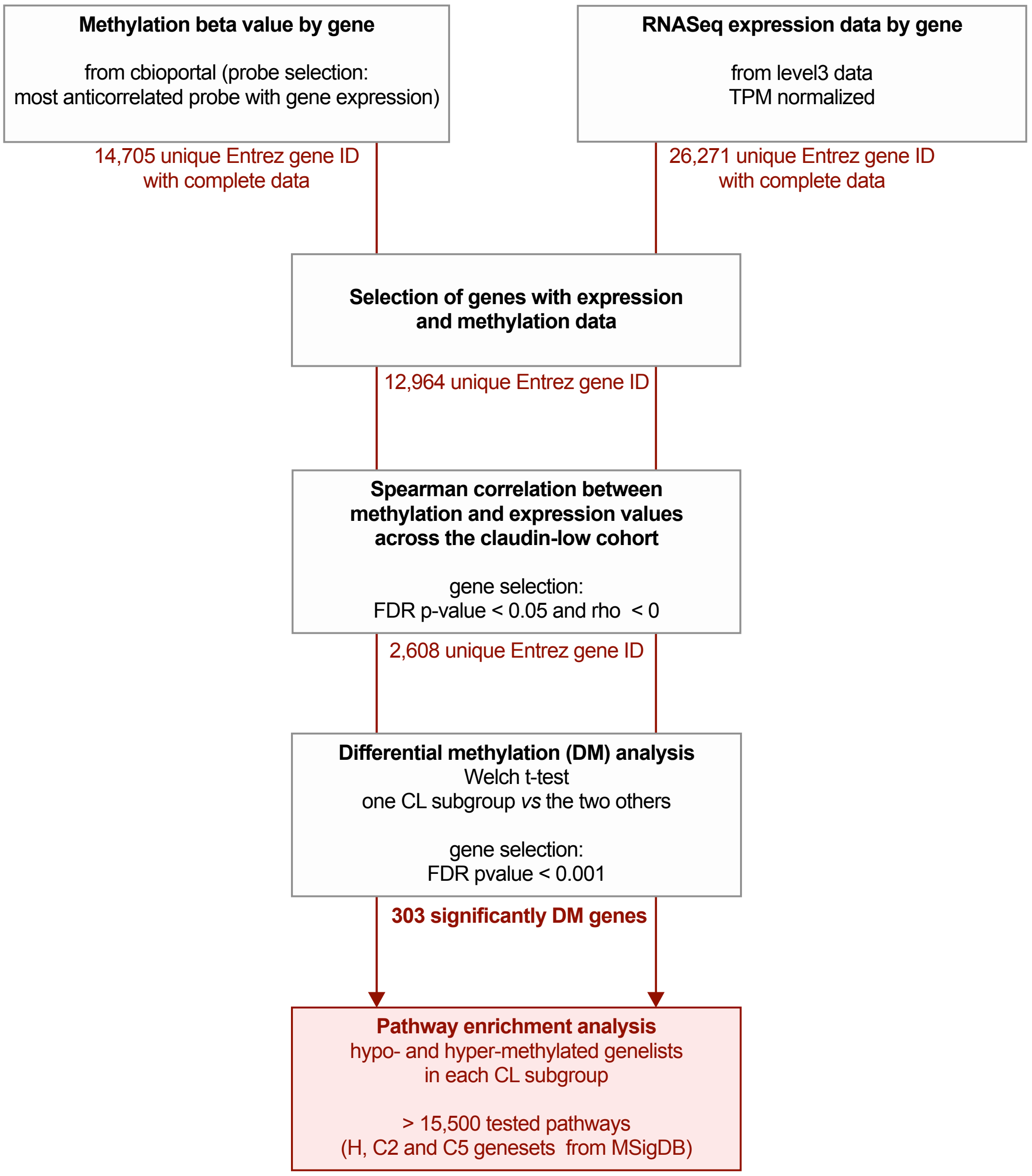

Supplementary Fig. 7: Methylation data analysis workflow

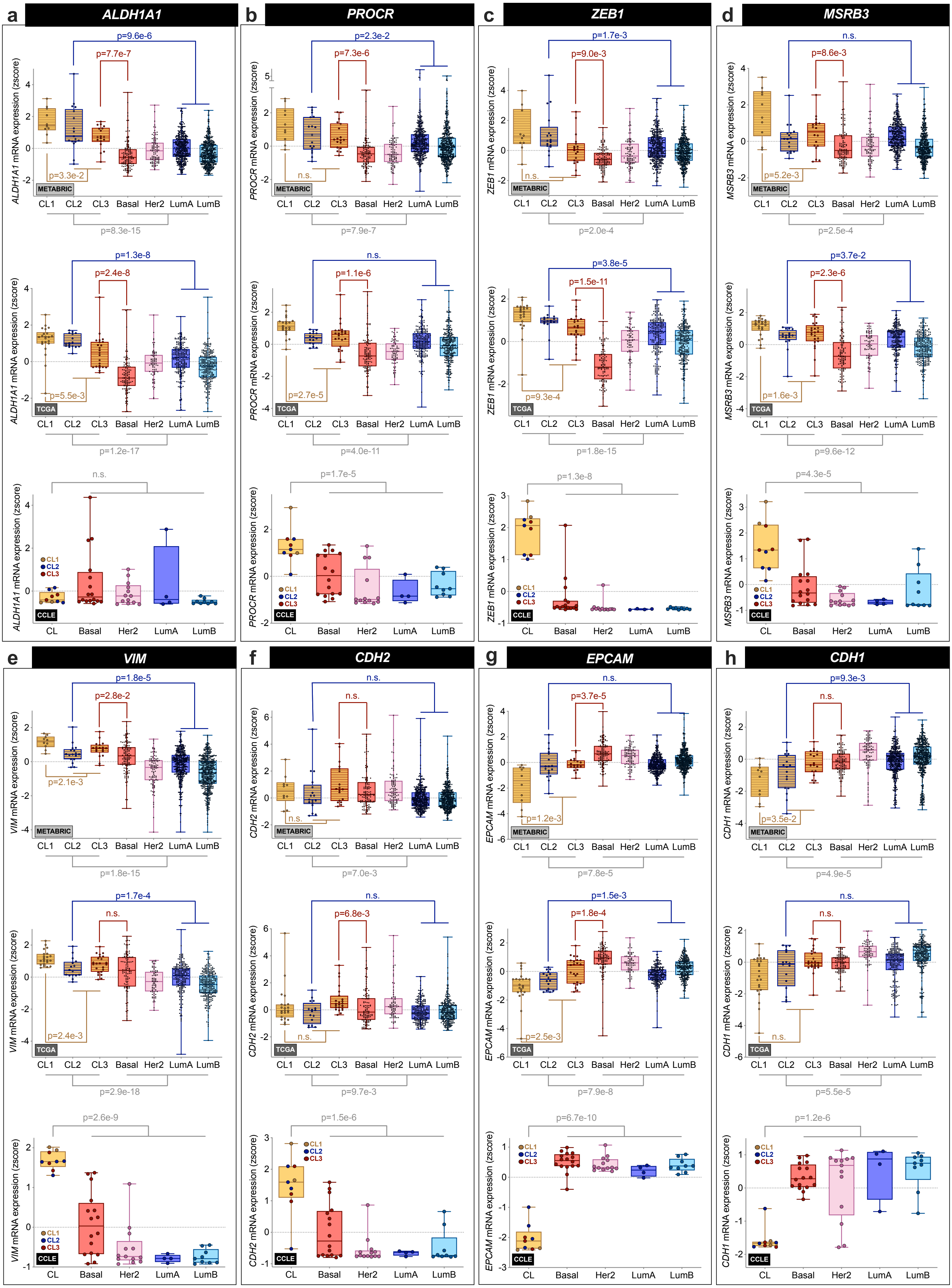

Supplementary Fig. 8: Gene expression of EMT and stemness markers
a-h- mRNA expression analysis of stemness and EMT gene markers per molecular subtype from the METABRIC cohort (top), the TCGA cohort (middle) and the CCLE database (bottom). METABRIC cohort: basal tumors (n=129), HER2 tumors (n=102), luminal A tumors (n=461), luminal B tumors (n=529), CL1 tumors (n=10), CL2 tumors (n=17), CL3 tumors (n=18); TCGA cohort: basal tumors (n=115), HER2 tumors (n=76), luminal A tumors (n=249), luminal B tumors (n=302), CL1 tumors (n=22), CL2 tumors (n=16), CL3 tumors (n=23); CCLE database: basal cell lines (n=16), HER2 cell lines (n=13), luminal A cell lines (n=4), luminal B cell lines (n=9), claudin-low cell lines (n=9). CL1 subgroup shows the highest stemness and EMT phenotype, while CL2 and CL3 display an intermediate stemness and EMT phenotype compared to their relative luminal/basal counterparts and CL1 tumors. Wilcoxon tests. Boxplot: center line, median; box limits, upper and lower quartiles; whiskers, minimum to maximum; all data points are shown

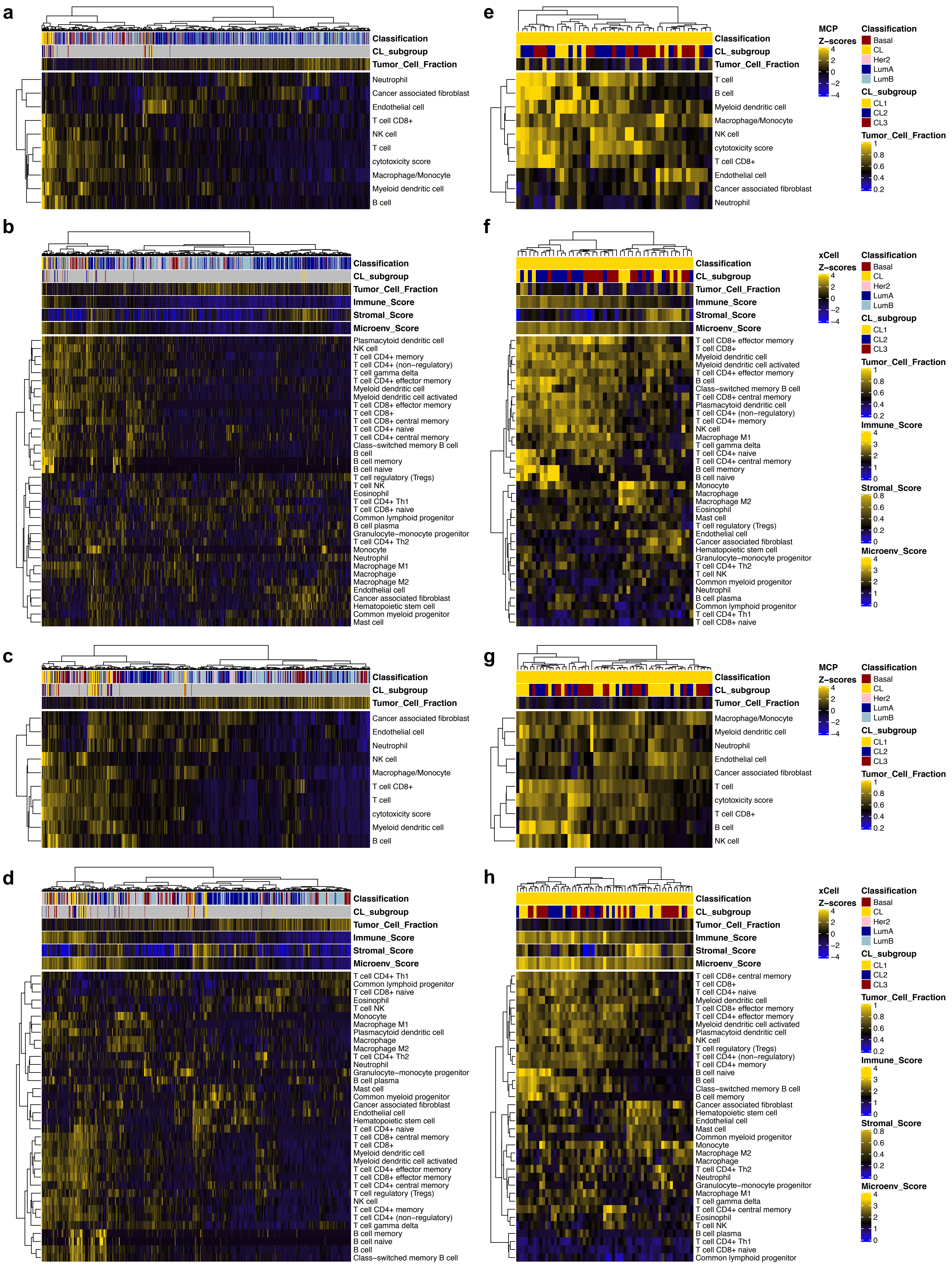

Supplementary Fig. 9: Microenvironment composition analysis

Heat-maps of gene expression signatures related to microenvironment composition for **a-d-** all breast cancer or **e-h-** claudin-low breast cancer samples from **a-b,e-f-** METABRIC or **c-d,g-h-** TCGA cohorts, using **a,e,c,g-** MCP counter or **b,d,f,h-** xCell algorithms. Clustering method: Ward’s; distance: Euclidean.

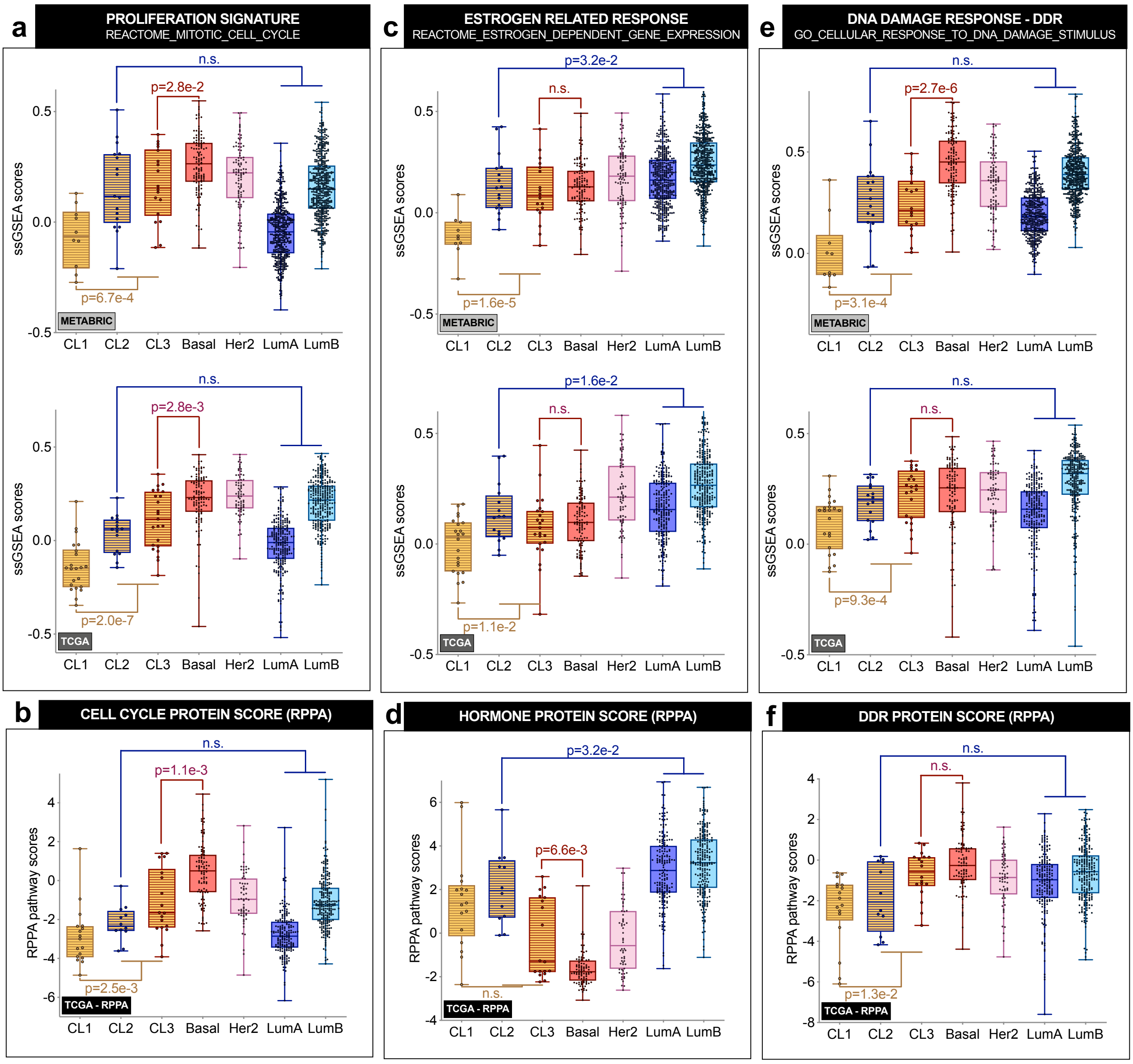

Supplementary Fig. 10: Gene expression and protein levels of proliferation, estrogen-related and DNA damage response pathways
a, c, e- ssGSEA score distribution for each molecular subtype, from the METABRIC cohort (top panel) and the TCGA cohort (bottom panel), related to **a-** proliferation, **c-** estrogen response and **e-** DDR pathways.). METABRIC cohort: basal tumors (n=129), HER2 tumors (n=102), luminal A tumors (n=461), luminal B tumors (n=529), CL1 tumors (n=10), CL2 tumors (n=17), CL3 tumors (n=18); TCGA cohort: basal tumors (n=115), HER2 tumors (n=76), luminal A tumors (n=249), luminal B tumors (n=302), CL1 tumors (n=22), CL2 tumors (n=16), CL3 tumors (n=23). **b, d, f-** Protein-based pathway scores (RPPA data) for each molecular subtype from TCGA breast tumors related to **b-** cell cycle, **d-** hormone and **f-** DDR pathways; TCGA cohort: basal tumors (n=96), HER2 tumors (n=62), luminal A tumors (n=188), luminal B tumors (n=245), CL1 tumors (n=17), CL2 tumors (n=12), CL3 tumors (n=18). Wilcoxon tests. Boxplot: center line, median; box limits, upper and lower quartiles; whiskers, minimum to maximum; all data points are shown. DDR: DNA damage response; RPPA: reverse phase protein array.

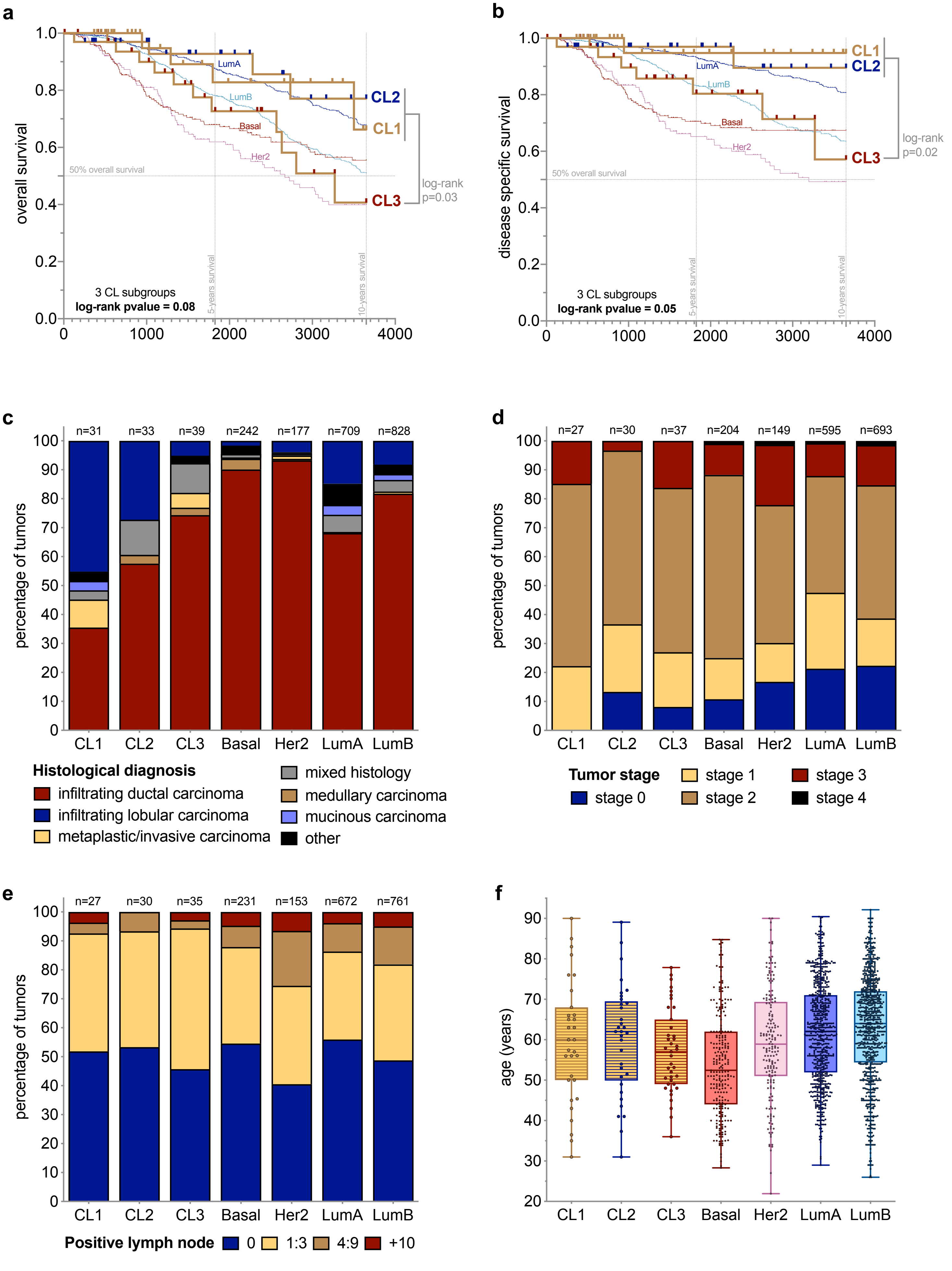

Supplementary Fig. 11: Clinicopathological features of claudin-low and non-claudin-low tumor subtypes

a- Overall survival, **b-** disease-specific survival, **c-** tumor histological subtype, **d-** tumor stage, **e-** lymph node involvement and **f-** patient age, according to their molecular subtypes from METABRIC and TCGA combined cohorts after tumor purity selection. Basal tumors (n=243), HER2 tumors (n=177), luminal A tumors (n=706), luminal B tumors (n=829), CL1 tumors (n=31), CL2 tumors (n=33), CL3 tumors (n=39). Boxplot: center line, median; box limits, upper and lower quartiles; whiskers, minimum to maximum; all data points are shown.
