# Supplementary material for: Comprehensive characterization of claudin-low breast tumors reflects the impact of the cell-of-origin on cancer evolution
Source: Nat Commun. 2020 Jul 9;11:3431. doi: 10.1038/s41467-020-17249-7 (PMC7347884; doi:10.1038/s41467-020-17249-7)
Supplement: Supplementary file 3 — Description of Additional Supplementary Files [file 41467_2020_17249_MOESM3_ESM.docx]

Description of Additional Supplementary Files

**Title:** Supplementary Dataset 1

**Description:** Differentially expressed genes along the mammary epithelial-cell hierarchy (MaSC1/2/3, LP and mL1/2) generated from transcriptomic data previously published in Morel et al., Nature Medicine 2017.
