# Supplementary material for: Comprehensive characterization of claudin-low breast tumors reflects the impact of the cell-of-origin on cancer evolution
Source: Nat Commun. 2020 Jul 9;11:3431. doi: 10.1038/s41467-020-17249-7 (PMC7347884; doi:10.1038/s41467-020-17249-7)
Supplement: Supplementary file 5 — Reporting Summary [file 41467_2020_17249_MOESM5_ESM.pdf]

nature research

Corresponding author(s): Alain PUISIEUX

Last updated by author(s): Jun 1, 2020

Reporting Summary

Nature Research wishes to improve the reproducibility of the work that we publish. This form provides structure for consistency and transparency in reporting. For further information on Nature Research policies, see Authors & Referees and the Editorial Policy Checklist.

Statistics

For all statistical analyses, confirm that the following items are present in the figure legend, table legend, main text, or Methods section.

n/a	Confirmed	
☐	☒	The exact sample size (*n*) for each experimental group/condition, given as a discrete number and unit of measurement
☒	☐	A statement on whether measurements were taken from distinct samples or whether the same sample was measured repeatedly
☐	☒	The statistical test(s) used AND whether they are one- or two-sided<br>*Only common tests should be described solely by name; describe more complex techniques in the Methods section.*
☒	☐	A description of all covariates tested
☐	☒	A description of any assumptions or corrections, such as tests of normality and adjustment for multiple comparisons
☒	☐	A full description of the statistical parameters including central tendency (e.g. means) or other basic estimates (e.g. regression coefficient) AND variation (e.g. standard deviation) or associated estimates of uncertainty (e.g. confidence intervals)
☐	☒	For null hypothesis testing, the test statistic (e.g. *F*, *t*, *r*) with confidence intervals, effect sizes, degrees of freedom and *P* value noted<br>*Give P values as exact values whenever suitable.*
☒	☐	For Bayesian analysis, information on the choice of priors and Markov chain Monte Carlo settings
☒	☐	For hierarchical and complex designs, identification of the appropriate level for tests and full reporting of outcomes
☐	☒	Estimates of effect sizes (e.g. Cohen's *d*, Pearson's *r*), indicating how they were calculated

Our web collection on statistics for biologists contains articles on many of the points above.

Software and code

Policy information about availability of computer code

Data collection	No software was used for data collection.
Data analysis	Figures were created using either the R software or GraphPad Prism 8.0 (GraphPad Software Inc., San Diego, USA).<br>All analyses were carried out with the R software (version 3.6.1) using following published R packages:<br>- heatmaps were generated with ‘ComplexeHeatmap’;<br>- principal component analysis was conducted with ‘ade4’;<br>- Gaussian finite models were performed with ‘mclust’ and ‘Rmixmod’;<br>- molecular breast subtype assignment were performed using the R package 'genefu';<br>- survival analyses were conducted using the R package 'survival';<br>- all pathway enrichment analyses were conducted using molecular signature database (MSigDB) gene sets H, C2 and C5 from ‘msigdbr’ R package, gene set enrichment analyses (GSEA) were carried out using ‘fgsea’ R package, single-sample GSEA (ssGSEA) scores were computed through ‘gsva’ R package;<br>- microarray data were RMA (robust multiarray average)-normalized through ‘oligo’ R package and differential expression analysis was performed using ‘limma’ R package;<br>- expression-based classifier for the three claudin-low subgroups was identified using shrunken nearest centroid method through ‘pamr’ R package;<br>- microenvironnement analysis was conducted using the R package 'Immunedeconv'.

For manuscripts utilizing custom algorithms or software that are central to the research but not yet described in published literature, software must be made available to editors/reviewers. We strongly encourage code deposition in a community repository (e.g. GitHub). See the Nature Research guidelines for submitting code & software for further information.

Data

Policy information about availability of data

All manuscripts must include a data availability statement. This statement should provide the following information, where applicable:

- Accession codes, unique identifiers, or web links for publicly available datasets
- A list of figures that have associated raw data
- A description of any restrictions on data availability

The complete set of CEL files from Morel et al. (Morel et al., Nature Medicine 2017) is available in the GEO database under accession number GSE56031 and the ArrayExpress database under accession number E-MTAB-4145.
METABRIC microarray expression data from discovery and validation sets were extracted from the EMBL-EBI archive (EGA, http://www.ebi.ac.uk/ega/; accession number: EGAS00000000083) ("Normalized expression data" files).
TCGA BRCA RNASeq expression data were extracted as FPKM values from the GDC data portal (https://portal.gdc.cancer.gov/).
RNASeq expression data from the CCLE breast cell lines were extracted as RPKM values from the CCLE data portal (https://portals.broadinstitute.org/ccle).
Triple negative (TN) status was determined from clinical data obtained through the Synapse platform (https://www.synapse.org/) (syn1757053) for METABRIC dataset and the GDC data portal (https://portal.gdc.cancer.gov/) for TCGA dataset.
METABRIC segmented copy-number data from discovery and validation sets were extracted from the EMBL-EBI archive (EGA, http://www.ebi.ac.uk/ega/; accession number: EGAS00000000083) ("Segmented (CBS) copy number aberrations (CNA)" files).
TCGA BRCA segmented copy-number data were extracted from the GDC data portal (https://portal.gdc.cancer.gov/).
ASCAT (Allele-specific copy number analysis of tumors) ploidy and purity estimates were extracted from COSMIC data repository (https://cancer.sanger.ac.uk/cosmic/).
Normalized methylation beta-values per gene were extracted from cBioPortal (http://www.cbioportal.org/).
Reverse phase protein assay (RPPA) level 4 data were extracted from the cancer proteome atlas (TCPA) portal (https://tcpaportal.org/tcpa/download.html).
METABRIC somatic mutation data from targeted sequencing were obtained from Pereira et al. (https://github.com/cclab-brca/mutationalProfiles/tree/master/Data) and TCGA somatic mutations data from whole exome sequencing were obtained from Ellrott et al..
Predicted IC50 data for MEK inhibitors (Trametinib, Selumetinib, Refametinib) were download from Genomics of drug sensitivity in cancer (GDSC) database (https://www.cancerrxgene.org/).
Complete clinical data were obtained through the Synapse platform (https://www.synapse.org/) (syn1757053) for METABRIC dataset.
For TCGA dataset, survival data were extracted from cBioPortal (Breast Invasive Carcinoma, TCGA, PanCancer Atlas) (http://www.cbioportal.org/) and other clinical data were obtained from the GDC data portal (https://portal.gdc.cancer.gov/).

Field-specific reporting

Please select the one below that is the best fit for your research. If you are not sure, read the appropriate sections before making your selection.

☒ Life sciences ☐ Behavioural & social sciences ☐ Ecological, evolutionary & environmental sciences

For a reference copy of the document with all sections, see nature.com/documents/nr-reporting-summary-flat.pdf

Life sciences study design

All studies must disclose on these points even when the disclosure is negative.

Sample size	All available breast samples from TCGA (n=1095), METABRIC (n=1992) and CCLE (n=51) databases were processed.
Data exclusions	In order to avoid any substantial bias due to non-tumor cell contamination, tumors without available estimation of ASCAT aberrant tumor cell fraction were removed from the whole cohort. Furthermore, a stringent purity threshold (ASCAT purity > 0.38) was determined, by applying Wilcoxon test, to select purest tumor samples from TCGA and METABRIC databases when applicable. The exclusion criteria was not pre-established but defined by the analysis at the beginning of the study as described in Fig.S1.
Replication	N/A
Randomization	All available breast samples from TCGA, METABRIC and CCLE databases were processed. No randomization was thus needed in this study.
Blinding	All available breast samples from TCGA, METABRIC and CCLE databases were processed. No blinding was thus needed in this study.

Reporting for specific materials, systems and methods

We require information from authors about some types of materials, experimental systems and methods used in many studies. Here, indicate whether each material, system or method listed is relevant to your study. If you are not sure if a list item applies to your research, read the appropriate section before selecting a response.

Materials & experimental systems

n/a	Involved in the study
☒	☐ Antibodies
☒	☐ Eukaryotic cell lines
☒	☐ Palaeontology
☒	☐ Animals and other organisms
☒	☐ Human research participants
☒	☐ Clinical data

Methods

n/a	Involved in the study
☒	☐ ChIP-seq
☒	☐ Flow cytometry
☒	☐ MRI-based neuroimaging
